# *Arabidopsis thaliana MLK3*, a Plant-Specific Casein Kinase 1, Negatively Regulates Flowering and Phosphorylates Histone H3 *In Vitro*

**DOI:** 10.3390/genes11030345

**Published:** 2020-03-24

**Authors:** Junmei Kang, Huiting Cui, Shangang Jia, Wenwen Liu, Renjie Yu, Zhihai Wu, Zhen Wang

**Affiliations:** 1 Institute of Animal Science, The Chinese Academy of Agricultural Sciences, Beijing 10019, China; kangjunmei@caas.cn (J.K.); wenwenliu3412@163.com (W.L.); 2 College of Grassland Science and Technology, China Agricultural University, Beijing 100193, China; Cuihting@163.com (H.C.); jsg200803@163.com (S.J.); 3 College of Life Sciences, Engineering Research Center of the Chinese Ministry of Education for Bioreactor and Pharmaceutical Development, Jilin Agricultural University, Changchun 130118, China; YRJ15843018186@163.com; 4 College of Agronomy, Jilin Agricultural University, Changchun 130118, China; wuzhihai1116@163.com

**Keywords:** *Arabidopsis thaliana*, casein kinase I, flowering, histone H3, *MLK3*, phosphorylation

## Abstract

*Arabidopsis thalianaMUT9-LIKE KINASES* (*MLKs*), a family of the plant-specific casein kinase 1 (CK1), have been implicated collectively in multiple biological processes including flowering. Three of the four *MLKs* (*MLK1/2/4*) have been characterized, however, little is known about *MLK3*, the most divergent member of *MLKs.* Here, we demonstrated that disruption of *MLK3* transcript in *mlk3* caused early flowering with retarded leaf growth under long-day conditions. *In vitro* kinase assay showed the nuclear protein MLK3 phosphorylated histone 3 at threonine 3 (H3T3) and mutation of a conserved residue (K146R) abolished the catalytic activity. Ectopic expression of *MLK3* but not *MLK3*(*K146R*) rescued the morphological defects of *mlk3*, indicating that an intact MLK3 is critical for maintaining proper flowering time. Transcriptomic analysis revealed that the floral repressor *FLOWERING LOCUS* C (*FLC*) was down-regulated significantly in *mlk3*, suggesting that *MLK3* negatively regulates flowering. Hence, *MLK3* plays a role in repressing the transition from vegetative to reproductive phase in *A. thaliana*. This study sheds light on the delicate control of flowering time by *A. thaliana CK1* specific to the plant kingdom.

## 1. Introduction

Casein kinase I (CK1) is a highly conserved serine/threonine-selective enzyme present in most eukaryotes. The CK1 encoding genes, which are ubiquitously expressed, play diverse cellular roles in eukaryotic organisms from yeast to humans [1,2]. Compared with animals, plants possess a large number of *CK1* [3]. For example, in angiosperm, two, five and 15 *CK1s* have been annotated in *Chlamydomonas reinhardtii*, *Selaginella moellendorffii* and *Oryza sativa*, respectively [4], suggesting an evolutionary expansion of *CK1* from lower plants to higher ones. The plant *CK1* phylogenetically splits into two subgroups: *CK1-like* (*CKL*) subgroup and a plant-specific *CK1* subgroup containing members from plant species exclusively. *Arabidopsis thaliana* genome encodes 13 *CKLs* [5] and four *CK1s* specific to the plant kingdom, which were named *MUT9-LIKE KINASEs* (*MLK1-4*) after the founding member *Mut9* in *C. reinhardtii* [6,7].

In the last decade, lots of progress has been made in unveiling the biological functions of *MLKs* and *MLK* homologs in crops as reviewed recently [4,8]. Previously, we found that the *mlk1 mlk2* double mutant displayed pleiotropic defects including dwarfism and hypersensitivity to osmotic stresses [6], suggesting the essentiality for plant growth and stress response. *MLKs*, also designated *Photoregulatory Protein Kinases* (*PPKs*), have been reported to implicate in the fine-tuning of plant response to solar radiation by coordinating the co-action of phytochrome and cryptochrome [9]. At the presence of phytochrome B (phyB), MLKs were co-purified with the circadian clock component EARLY FLOWERING 3 (ELF3)/ELF4 by affinity purification [10]. Consistently, Ni and coauthors found that MLKs interacted and phosphorylated both phyB and phytochrome-interacting factor3 (PIF3) [11]. MLKs were also shown to catalyze phosphorylation of the blue-light receptor cryptochrome 2 (CRY2), which was an interacting partner of MLKs [9]. The physical structure analysis of MLKs revealed that the conserved N-terminal domain and the non-kinase domain at C-terminal facilitated interaction with PIF3 and CRY2 in response to red and blue light, respectively [9,11]. Recently, MLKs, also described as *A. thaliana* EL1-like (AEL) proteins, were documented to phosphorylate ABA signal receptors PYRABACTIN RESISTANCE/PYR-LIKE (PYR/PYL) proteins to promote degradation by ubiquitination, and the triple mutants of *mlks* were hypersensitive to ABA treatment due to eliminated ABA responses [12]. One of the triple mutants, *mlk1,3,4*, was also detected with elevated level of defense marker genes induced by salicylic acid (SA), and MLKs were identified as interacting proteins with RADICAL-INDUCED CELL DEATH1 (RCD1), a positive regulator of SA signaling [13]. Taken together, *MLKs* collectively play critical roles in Arabidopsis growth and development by affecting diverse biological processes, such as stress response, circadian rhythm, light signaling and immunity. 

*MLKs*, together with rice ortholog *Early Flowering 1* (*EL1*)/*Heading Date 16* (*Hd16*), have been shown to involve in flowering regulation. Mutation of *EL1*/*Hd16* caused early flowering of 5–6 days in comparison with wild type [14]. *EL1/Hd16* acts as a multifunctional kinase to phosphorylate hierarchical flowering-regulating proteins, including DELLA protein SLENDER RICE1 (SLR1), circadian clock component PSEUDO-RESPONSE REGULATOR 37 (OsPRR37) and floral repressor GRAIN NUMBER, PLANT HEIGHT, AND HEADING DATE 7 (Ghd7) [14,15,16]. In *A. thaliana*, *mlk4* and the higher order of *mlk4*-combined mutants flowered late [10]. Recently, MLK4 has been reported to interplay with circadian clock component CIRCADIAN CLOCK-ASSOCIATED 1 (CCA1) to affect the expression of *GIGANTEA* (*GI*), a positive flowering regulator [17], suggesting *MLK4* regulates flowering probably via circadian rhythm. This study focused on the function of *MLK3*, the most divergent *member of MLKs*, in flowering by investigating an *mlk3* mutant allele with disrupted *MLK3* transcript. Our molecular, histochemical and genetic findings demonstrated that MLK3 phosphorylated histone H3 at threonine 3 *in vitro*, and disruption of *MLK3* led to the down-regulation of *FLOWERING LOCUS* C (*FLC)* and early flowering, which could be rescued by constitutive expression of *MLK3*. These results suggest that *MLK3* plays a negative role in *A. thaliana* flowering.

## 2. Materials and Methods

### 2.1. Plant Materials and Growth Conditions

*A. thaliana* seeds of the T-DNA insertion line (SALK_017102) were obtained from the Arabidopsis Biological Resource Center [6] and ecotype Col-0 was used as wild type. Mature seeds were imbibed in water and treated at 4 °C for two days before germination in soil (a standard potting compost (M3) mixed with perlite and vermiculite at 3:1:1). Plants were grown under normal conditions (21 °C) at long-day (LD) (16 h light/8 h dark) with light supplied at 100 µmol/m^2^/s, or short day (8 h light/16 h dark) conditions with the same light intensity. 

### 2.2. Plasmid Constructs and Plant Transformation

For the *35S::MLK3* construct, *MLK3* CDS was amplified from reverse-transcribed cDNA. The sequence-verified PCR product was ligated into pROK2 vector after digestion by *Bam*H I and *Sac* I. For point mutation, the oligonucleotide primers containing the desired mutation of *MLK3* CDS were used according to the instruction of QuikChange II Site-Directed Mutagenesis kit (Agilent Technologies, Palo Alto, CA, USA). Primer sequences were listed in Table S1.

### 2.3. In Vitro Protein Kinase Activity Assay 

The open reading frame (ORF) of *MLK3* without the nucleotide sequence (TGA) for the translation stop codon was amplified by reverse transcription-polymerase chain reaction (RT-PCR). The verified amplicon was cloned into *Not* I and *Bam*H I sites of vector pMAL-c5X (NEB). For the generation of MLK3 (K146R)-MBP, the point-mutated *MLK3* plasmid was used as a template with the same primers for MLK3-MBP. After sequence verification, PCR product was digested by *Not* I and *Bam*H I, then ligated into pMAL-c5X. The MBP-fused protein was expressed in *E. coli* BL21 (DE3) strain. The recombinant protein was purified with Amylose Resin (NEB) after induction for 16 hours with IPTG (0.1 mM). Protein kinase activity was assayed by dot blotting with antibody against H3T3 phosphorylation (Upstate, 07–424) as described [6]. An unmodified histone H3 peptide (residues 1-21) biotin conjugate (Upstate 12-403) was used as a substrate.

### 2.4. mRNA Sequencing and Data Analysis

For mRNA-sequencing, rosette leaves from 11-day-old plants grown under LD were used to isolate total RNA with TRIzol reagent (Invitrogen, Carlsbad, CA, USA). mRNA library was prepared according to the Illumina’s protocol (mRNA-seq Lib Prep Kit RK20302). Three independent replicates were performed. The libraries were sequenced using a Genome Analyzer IIx (Illumina, San Diego, CA, USA). The transcription analysis was processed with a regular RNA-sequencing workflow on bioconductor. Briefly, the reference genome of *A. thaliana* (ftp://ftp.ensemblgenomes.org/pub/plants/release-42/fasta/arabidopsis_thaliana/dna/) was used to map the clean reads after trimming the raw read data by TopHat 2.1.1 [18]. Gene expression values were calculated and differentially expressed genes were determined by DESeq2 package [19]. Gene Ontology plotting was performed using Bioconductor packages ggplot2, clusterProfiler [20] and org.At.tair.db. 

### 2.5. Immunoblot Analysis

For dot blotting, reactions containing the recombinant protein MLK3-MBP or MLK3 (K146R)-MBP, substrate (250 ng of an unmodified histone H3 peptide), phosphatase inhibitor (Roche Applied Science) and/or ATP were carried out at 30 °C (60 min). One µl of the reaction mixture was spotted on a nitrocellulose filter and air-dried. Ponceau S stained membrane was scanned to indicate protein loading and then applied to regular immunoblotting as described [6]. For western blot, nuclear proteins were isolated from two-week-old plants as reported [21] and separated on 15% SDS-PAGE before electroblotting onto nitrocellulose membrane. Antibody against H3T3 phosphorylation (Upstate, 07–424) was used with a modification-insensitive anti-H3 antibody (Abcam, ab1791) as an internal loading control. 

### 2.6. Subcellular Localization and Nuclear Staining

To generate the MLK3-GFP fusion protein, the CDS of *MLK3* was cloned into pENTR/D-TOPO (Life Technologies, Carlsbad, CA, USA) and recombined into destination vector pK7FWG2.0 using Gateway LR Clonase II Enzyme Mix (Life Technologies). The *Agrobacterium tumefaciens* expressing *35S::GFP* or *35S::MLK3-GFP* were separately infiltrated into tobacco leaf with needle-free syringe. Subcellular localization was examined using a Zessi confocal laser scanning microscope (Zeiss Axioskop, Germany) 36 h after infiltration. VECTASHIELD^®^ with DAPI (Vector labs H-1200) was used to stain the nucleus before capturing the image.

## 3. Results

### 3.1. MLK3 Belonged to a Subgroup Divergent from Its Paralogs

**Figure 1:**
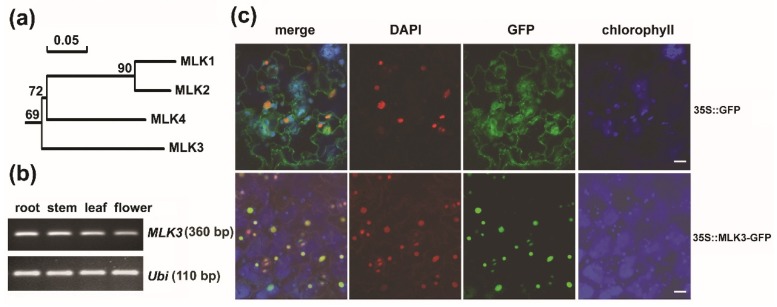
Phylogenetic analysis and expression pattern of *MLK3*. **(a)** Phylogenetic analysis of the four MLKs using DNAMAN (version 7.0, Lynnon Corporation, Quebec, Canada). (**b**) Expression analysis of *MLK3* transcript by reverse transcription-polymerase chain reaction (RT-PCR). Total RNA of roots and leaves was isolated from 2-week-old plants, and total RNA of stems and flowers was from mature plants. *POLYUBIQUITIN 10* (*At4g05320*) was used as an internal standard. (**c**) Subcellular localization of the MLK3-GFP fusion protein in tobacco leaf. Agrobacterium expressing *35S::MLK3-GFP* or *35S::GFP* was infiltrated into tobacco leaves separately. Transiently expressed GFP was imaged by confocal fluorescence microscopy 36 hours after the infiltration. Nucleus was indicated by DAPI staining. Bars = 10 µm.

In *A. thaliana*, four plant-specific casein kinase 1 encoding genes named *MLK1-4* have been reported [6]. The four MLKs shared sequence identity of 67.9%–91.1%. Homology analysis of MLKs and the homologs from seven crops demonstrated that the plant-specific CK1s were subgrouped into two main branches (I and II). Divergent from its paralogs clustered into Branch I, MLK3 belonged separately to Branch II (Figure S1). The phylogenetic analysis showed that MLK3 was relatively distant from other three MLKs (Figure 1a). Sequence alignment showed that MLK3 shared the conserved CK1 functional domains including substrate recognition region, kinase catalytic loop, ATP binding site and a predicted nuclear localization signal [7] (Figure S2). MLK3 and its paralogs have an isoelectric point ranging from 9.09 to 9.66 (Table S2), suggesting the preference to acidic substrates, such as serine and threonine residues. Thus, albeit divergent from other MLKs, MLK3 shares the common features of CK1, implying its enzymatic activity as a kinase in protein phosphorylation. 

### 3.2. MLK3 Was a Nuclear Protein and MLK3 Was Expressed Ubiquitously

Three of the four *MLKs* (*MLK1*, *2* and *4*) have been functionally identified in in recent years [6,17,22]. To examine the spatial and temporal expression patterns of *MLK3*, we performed semi-quantitative RT-PCR. As shown in Figure 1b, *MLK3* transcript was detected in roots, stems, leaves and flowers, which is in agreement with the results from eFP Browser (http://bbc.botany.utoronto.ca/efp) [23]. The results suggest that CK1 encoding gene *MLK3* is expressed ubiquitously in *A. thaliana* tissues.

Considering that MLK3 has a predicted nuclear localization signal (Figure S2) [6,7], the subcellular localization of the MLK3-GFP recombinant protein was examined. As expected, when transiently expressed in tobacco leaves by infiltration, the green signal of the MLK3-GFP fusion protein was observed exclusively in the nucleus of the leaf epidermal cells as indicated by DAPI staining. In contrast, the signal of 35S::GFP displayed a universal distribution in the epidermal cells (Figure 1c). Consistent with the previous findings in tobacco and *A. thalia*na protoplasts [9,10,12], MLK3 is a nuclear protein, implying its potential role in histone modification.

### 3.3. MLK3 Phosphorylated Histone H3 and an Intact Lysine (K146) Was Required for In Vitro Activity

**Figure 2:**
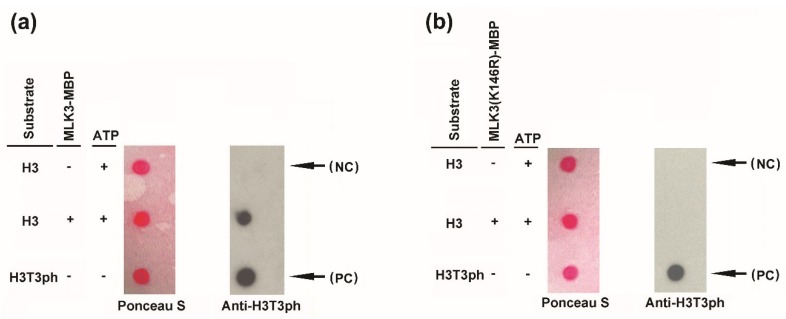
*In vitro* kinase activity assay of MLK3. The kinase activity assay of the recombinant protein MLK3-MBP **(a)** and MLK3 (K146R)-MBP **(b)** by dot blotting. MLK3 and MLK3 (K146R) were fused with maltose-binding protein (MBP) separately. H3, an unmodified histone H3 peptide (residues 1-21), biotin conjugate (Upstate 12-403) was used as a substrate. The reaction mixture (1 µl) was spotted on a nitrocellulose membrane as indicated by Ponceau S staining and phosphorylation was examined by immunoblotting with an antibody against H3T3ph (Upstate, 07–424). The reaction mixture without the recombinant protein(s) served as the negative control (NC) and peptide H3T3ph, phospho-histone H3 (Thr3) peptide (residues 1–21), biotin conjugate was used as the positive control (PC).

Given that the nuclear protein MLK3 shares the canonical features of CK1, we tested whether MLK3 functions as a protein kinase. First, MLK3 was fused with maltose-binding protein (MBP) and expressed in *E. coli* BL21 (DE3) strain. The purified MLK3-MBP recombinant protein was then incubated with the phosphoryl donor ATP and substrate before being dotted on the membrane. Finally, immuno-blotting was performed using an antibody specifically against phosphorylated H3T3 [7]. The results showed that strong immune-signal (bottom row) was detected with H3T3ph peptide (a phosphor-histone H3 (Thr3) peptide) (Figure 2a), which was used as the positive control, confirming the specificity of the antibody. For the substrate of an unmodified histone H3 peptide (H3), anti-H3T3ph signal was detected when both ATP and the recombinant protein MLK3-MBP were present (middle row) (Figure 2a). In the absence of the MLK3-MBP fusion protein, anti-H3T3ph signal was undetectable (upper row) (Figure 2a). The detection of the phosphorylated histone H3 at threonine 3 (H3T3ph) indicated that with ATP as phosphoryl donor, the recombinant MLK3-MBP protein catalyzed *in vitro* phosphorylation of the unmodified histone H3 peptide. Hence, MLK3 phosphorylated histone H3T3, a predominant target of the plant-specific kinase Mut9 and MLK1 in *C. reinhardtii* and *A. thaliana*, respectively [6,7]. 

It has been reported that the conserved lysine residue (K174 for Mut9p, K175 for MLK4/PPK1) was essential for the catalytic activity [7,9,24]. To test whether the counterpart lysine (K146) of MLK3 is critical for H3T3 phosphorylation, the conserved K146 was point mutated to arginine (R). The MBP-fused MLK3 (K146R) was purified from *E. coli.* for the kinase activity assay as described above. Anti-H3T3ph signal was detected with H3T3ph peptide (the positive control), but no immuno-signal was detectable for the unmodified histone H3 peptide either with or without the recombinant protein MLK3 (K146R)-MBP (Figure 2b). These results indicated that unlike MLK3, the point-mutated MLK3 (K146R) was catalytically inactive, suggesting that an intact lysine at the conserved position is crucial for MLK3 to phosphorylate substrate proteins.

### 3.4. MLK3 Affected Leaf Growth and Flowering Time

**Figure 3:**
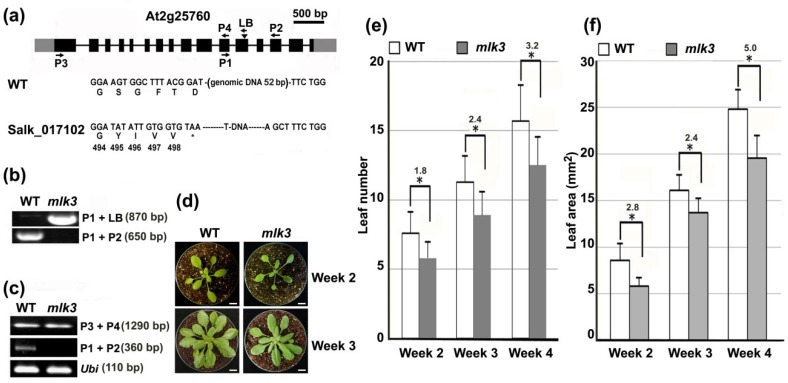
Leaf growth was retarded in *mlk3*. (**a**) Schematic representation of *MLK3* (*At2g25760*) locus with a T-DNA insertion. Introns, exons and un-translated regions were represented as lines, black boxes and gray boxes, respectively. Triangle indicated the T-DNA insertion site of *mlk3* (SALK_017102). Arrow indicated the location of primers (LB and P1-P4) used for genotyping or *MLK3* transcript detection. Star represented the translation stop codon. (**b**) Genotyping of *mlk3* by PCR with the indicated primer combinations. (**c**) Transcriptional analysis of *MLK3* by RT-PCR using primers of P1 & P2 or P3 & P4 as indicated in (**a**). *POLYUBIQUITIN 10* was used as an internal standard. (**d**) Image of the representative plants at week-2 (upper panel) and week-3 (lower panel). Bar = 1 cm. (**e**) Comparison of the leaf number between wild type and *mlk3* at the indicated time points. * indicated the significant difference from wild type (Student’s *t*-test, *p* < 0.05). Leaf number difference between the two genotypes was indicated. Leaf with petiole was counted from 15 plants of each genotype and three replicates were conducted independently. (**f**) Comparison of leaf area between wild type and *mlk3* at the indicated time points. The 5th leaf was measured and the leaf area was calculated using ImageJ (https://imagej.nih.gov/ij/download.html). Three batches of plants with 15 plants per batch were analyzed independently. * indicated the significant difference from wild type (Student’s *t*-test, *p* < 0.05). The difference of leaf area between the two genotypes was indicated (mm^2^).

To address the biological function of *MLK3*, a T-DNA insertion line (SALK_017102) was obtained [6]. PCR analysis revealed that the T-DNA was integrated into the 12^th^ exon of *MLK3* (Figure 3a,b). In homozygous *mlk3* mutant, *MLK3* transcript flanking the insertion site was undetectable by RT-PCR, while a transcript upstream of the insertion site was detected (Figure 3c), suggesting the partial expression of *MLK3*. Hereafter, the primers flanking the T-DNA insertion site were used to analyze the expression of *MLK3*. Morphologically, during the vegetative stage, *mlk3* was slightly smaller than wild type under LD (Figure 3d). Compared with wild type, the rosette leaf number of *mlk3* was fewer on average than wild type at weeks 2–4 (*p* < 0.05) (Figure 3e). Calculation of leaf area (the 5th leaf) of these plants demonstrated that the fifth leaf of *mlk3* was about 2.4–5.0 mm^2^ smaller than that of wild type (*p* < 0.05) (Figure 3f). The results suggest the progressive retardance of leaf growth in *mlk3*.

**Figure 4:**
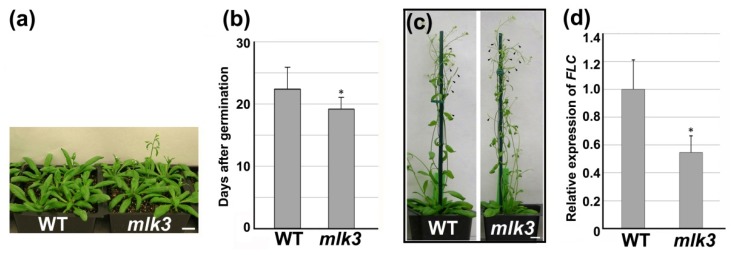
Disruption of *MLK3* caused early-flowering. (**a**) Representative image of 24-day-old plants grown under long-day (LD). Bar = 2 cm. (**b**) Flowering time analysis. Days after germination (DAG) were measured as the number of days from germination to the emergence of the first flower under LD. Plants (*n* ≥ 30) were counted from three independent replicates. * indicated the significant difference from wild type (Student’s *t*-test, *p* < 0.05). (**c**) Representative image of mature plants (five-week-old) under LD. Arrowhead indicated young siliques. Bar = 1 cm. (**d**) Relative expression level of *FLOWERING LOCUS* C (*FLC)* by qRT-PCR and *POLYUBIQUITIN 10* was used as the internal control. Three biological replicates were performed. * indicated the significant difference from wild type (Student’s *t*-test, *p* < 0.05).

For flowering time, in terms of days after germination (DAG), *mlk3* flowered at 19.2 DAG under LD, while wild type flowered at 22.3 DAG (*p* < 0.05) (Figure 4a,b), indicating that *mlk3* flowered about three-days earlier than wild type. This observation is consistent with the previous report that compared with wild type, *mlk3* needs a couple fewer days to have inflorescence of one centimeter [10]. Consequently, *mlk3* at week-5 displayed more siliques (16/plant vs 8/plant, *p* < 0.01) than wild type at the same stage (Figure 4c). No obvious difference in flowering time was observed between *mlk3* and wild type under short day (SD) conditions (Table S3). Therefore, disruption of *MLK3* altered leaf growth and flowering time simultaneously under LD. 

### 3.5. Disruption of MLK3 Repressed the Expression of FLC 

To profile the transcriptome of *mlk3*, RNA-sequencing was carried out as described [6]. Consistent with the result of Figure 3c, in *mlk3*, the unique reads matched to *MLK3* exons downstream of the T-DNA insertion site showed a clear depletion, while the reads upstream of the interruption were similar to those of wild type (Figure S3a). Hence, the *MLK3* transcript in *mlk3* is disrupted by T-DNA insertion. By the criteria of │Log_2_FC│ ≥ 1 and *p* < 0.01, a total of 549 genes were differentially expressed with 165 up-regulated and 384 down-regulated in *mlk3* relative to wild type (Figure S3b). Among them, the expression level of *FLC*, a negative flowering integrator, was decreased to 40% of wild type (Table S4). A similar result was obtained by qRT-PCR (Figure 4d). No significant change of *MLK3* paralogs was detected in *mlk3*, implying no clear compensation of other *MLKs*. None other known flowering regulators significantly altered the transcriptional level in *mlk3*. Thus, disruption of *MLK3* led to the down-regulation of *FLC*, which de-repressed flowering in *mlk3*. 

Previously, we showed that MLK1 and Mut9p were responsible for phosphorylation of histone H3T3 *in vivo* [6,7]. To compare the global level of H3T3ph between *mlk3* and wild type, western blot was performed with anti-H3T3ph antibody. The intensity of H3T3ph in *mlk3* was not notably different from that of wild type (Figure S4), suggesting the functional redundancy of other kinases, especially *MLK1* and *MLK2*, which contributed to *in vivo* phosphorylation of H3T3 in *A. thalianna* [6]. 

### 3.6. The Negative Role of MLK3 in Flowering Required the Intact Lysine (K) 146

**Figure 5:**
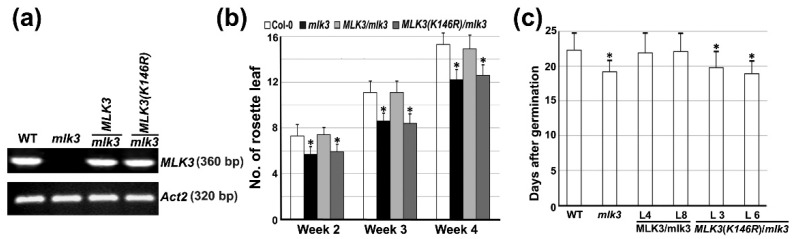
Analysis of leaf growth and flowering time in *mlk3* plants expressing *35S::MLK3* or *35S::MLK3(K146R)*. (**a**) Transcriptional analysis of *MLK3* by semi-quantitative RT-PCR. Total RNA was isolated from 10-day-old plants of the indicated genotypes. Line 8 and Line 6 were analyzed for the transgenic plants of *35S::MLK3/mlk3* and *35S::MLK3(K146R)/mlk3*, respectively, and *Actin 2* (*At3g18780*) was used as internal control. (**b**) Comparison of the rosette leaf number of the transgenic *mlk3* plants expressing *35S::MLK3* or *35S::MLK3(K146R)*. Transgenic Line 8 and Line 6 were used for *35S::MLK3/mlk3* and *35S::MLK3(K146R)/mlk3,* respectively. * indicated a significant difference from wild type (Student’s *t*-test, *p* < 0.05). Leaf with petiole was counted with at least ten plants for individual genotype and three replicates were conducted independently. (**c**) Flowering time analysis of the indicated transgenic plants under LD. Two independent transgenic lines were analyzed. * indicated significant difference from wild type (Student’s *t*-test, *p* < 0.05). Three biological replicates were performed with at least 15 plants in total.

To provide genetic evidence of *MLK3* in flowering regulation, *MLK3* CDS driven by the 35S promoter was introduced into *mlk3*. The transcriptional analysis of *MLK3* using semi-quantitative RT-PCR showed that unlike *mlk3*, in the transgenic *mlk3* plants expressing *35S::MLK3* (e.g., transgenic Line 8) *MLK3* transcript was detectable (Figure 5a). The transgenic line possessed a similar number of rosette leaves to that of wild type (Figure 5b). In terms of DAG, the two independent transgenic lines (Line 4 and Line 8) were not significantly different from wild type (Figure 5c). These results indicated that the constitutive expression of *MLK3* rescued the morphologic abnormalities of *mlk3* in both leaf growth and flowering time. 

To determine whether an intact K146 is critical for *MLK3*-mediated flowering, *35S::MLK3 (K146R)* was introduced into *mlk3*. The transcriptional analysis showed a similar intensity of *MLK3* transcript in the transgenic plant (e.g., transgenic Line 6) to that of wild type (Figure 5a). On the contrary, the leaf number of the transgenic *mlk3* ectopically expressing *MLK3 (K146R)* was significantly fewer than wild type, showing a similar number of leaves to that of *mlk3* (Figure 5b). Flowering time analysis of two independent transgenic lines (lines 3 and 6) demonstrated that similar to *mlk3*, DAG of both transgenic lines was significantly fewer than wild type (*p* < 0.05) (Figure 5c), suggesting that the transgenic lines flowered earlier. The results indicated that unlike *MLK3*, which successfully restored the phenotypic abnormalities of *mlk3*, the catalytically inactive *MLK3 (K146R)* had no clear effect on flowering or leaf growth. Therefore, the conserved lysine K146 essential for phosphorylation of H3T3 is indispensable for *MLK3*-mediated flowering repression. 

## 4. Discussion

Casein kinase 1 (CK1) is a conserved ser/thr protein kinase family universally present in eukaryotic organisms [1]. In mammals, six CK1 isoforms (alpha, beta, gamma, delta and epsilon) have been reported to involve in a variety of cellular processes such as chromosome segregation and cellular differentiation by phosphorylating a wide range of substrates [1]. The plant kingdom possesses a unique clade of *CK1* evolutionarily related but phylogenetically distinct from mammalian *CK1*. In angiosperm, model species *C. reinhardtii, A. thaliana* and *O. sativa* have two, four and six *CK1* members exclusive to plants, respectively [4,7,15,16]. The expansion of the plant-specific *CK1* may be attributed to the sessile lifestyle. 

It appears that the intact kinase activity of the plant-specific CK1, especially the MLK family members is indispensable for plant growth and development. Albeit relatively divergent from its paralogs, MLK3 shares the common functional domains with a sequence identity ranging from 67.9% to 72.7% [6,7]. As expected, the nuclear protein MLK3, like its homolog MUT9 and MLK1, *in vitro* phosphorylated histone H3 at threonine 3 (H3T3) [6,7]. Mutation of a conserved lysine residue (K146) abolished the enzymatic activity of MLK3(K146R). This is consistent with the eliminated catalytic activity of Mut9 and MLK4/PPK1 when the counterpart lysine, i.e., K174 for *C. reinhardtii* Mut9 and K175 for *A. thaliana* MLK4/PPK1, was mutated [7,9]. In agreement with the *in vitro* findings, the constitutive expression of *MLK3* but not the point-mutated *MLK3 (K146R)* complemented the defects of *mlk3*. These findings suggest that the plant-specific CK1s share a similar catalytic structure and probably mechanism in phosphorylating target proteins. 

The plant-specific *CK1* implicates in the transition from vegetative to reproductive stage [16,17]. Mutants of several *MLK*-family members in rice and Arabidopsis displayed abnormal flowering phenotype. For example, the rice mutant *el1*, which was deficient in a plant-specific casein kinase I, exhibited enhanced gibberellin (GA) response and early flowering with slow leaf emergence rate [16]. Consistently, *Hd16*, the identical gene of *EL1*, was confirmed as a flowering-time quantitative trait locus [15]. *A. thaliana* loss-of-function mutant of *MLK4* and the higher order of *mlk4*-combined mutants flowered late [10]. We demonstrated that the disruption of *MLK3* slightly accelerated flowering and ectopic expression of *MLK3* rescued the phenotype, indicating the negative role of *MLK3* in *A. thaliana* flowering. Therefore, *MLK3,* together with its homologs, plays a critical role in flowering regulation.The plant-specific CK1 regulates flowering by modulating diverse substrates including histones and flowering components of multiple signaling pathways, such as light signaling, circadian clock and GA. For example, MLK4 *in vitro* phosphorylated histone H2A on serine 95 although in late-flowering *mlk4* mutant the level of phosphorylated H2A S95 was not significantly different from that of wild type [17]. Our results that MLK3 phosphorylated histone H3T3 *in vitro* and mutation of the kinase activity failed to rescue the early-flowering phenotype of *mlk3* suggest that MLK3-mediated phosphorylation of H3T3ph is crucial for *A. thaliana* flowering. In addition to histone proteins, recent studies on *mlk3/ppk4*-*combined MLKs/PPKs* triple mutants revealed that MLK3/PPK4, together with its paralogs, targeted light signaling receptors or coordinators. For instance, the level of phosphorylated PIF3, a negative regulator of flowering, was reduced in red-light hypersensitive triple mutant *ppk124* and *ppk134* [11,25], suggesting that normal light-induced PIF3 phosphorylation required the *MLKs/PPKs* collectively. The inhibition role of *MLK* homologs in flowering was first reported in rice, and EL1/Hd16 was found to specifically phosphorylate DELLA protein SLR1, which was required for the negative effect of SLR1 on GA signaling [16]. Moreover, EL1/Hd16-mediated phosphorylation of Ghd7 (Grain number, plant height, and heading date 7) enhanced photoperiod response. Taken together, these findings support the notion that *MLK3,* together with its homologs, is involved in flowering regulation by phosphorylating a diverse spectrum of proteins. Further study on loss-of-function mutant would provide direct evidence of *MLK3* in *A. thaliana* flowering regulation. Future investigation of the specificity of individual *MLKs*, especially *MLK3* and *MLK4* in *A. thaliana* flowering may shed light on the delicate control of shift from vegetative to reproductive phase by the plant-specific *CK1*. 

## 5. Conclusions

As serine/threonine kinases, *A. thaliana MLKs* belong to a subgroup of casein kinase 1 specific to the plant kingdom. Interruption of *MLK3,* the most divergent member of *MLKs*, caused early flowering with decreased *FLC* transcript, suggesting that *MLK3* negatively regulates flowering probably by de-repressing *FLC*. The nucleus-localized MLK3 catalyzed the phosphorylation of histone H3 at threonine 3 *in vitro,* and an intact lysine residue (K175) was critical for the catalytic activity of MLK3 and for the maintenance of proper flowering time. This study provides important evidence that the plant-specific CK1 plays a key role in flowering regulation.

## Supplementary Materials

The following are available online at https://www.mdpi.com/2073-4425/11/3/345/s1, **Figure S1**: Homology analysis of the plant-specific CK1s in the indicated higher plants; **Figure S2**: Sequence alignment of MLK1-4 in Arabidopsis; **Figure S3:** Transcriptome analysis of *mlk3* mutant relative to wild type; **Figure S4**: Analysis of H3T3ph level by western blot; **Table S1**: Sequences of the primers used in the study; **Table S2**: Basic information of the deduced MLK1-4 proteins in Arabidopsis; **Table S3**: Flowering time analysis of *mlk3* under short-day conditions; **Table S4**: The differentially expressed genes in *mlk3* mutant.
