# Supplementary material for: Arabidopsis thaliana MLK3, a Plant-Specific Casein Kinase 1, Negatively Regulates Flowering and Phosphorylates Histone H3 In Vitro
Source: Genes (Basel). 2020 Mar 24;11(3):345. doi: 10.3390/genes11030345 (PMC7141126; doi:10.3390/genes11030345)
Supplement: Supplementary file 1 [file genes-11-00345-s001.zip › Additional files R1.pdf]

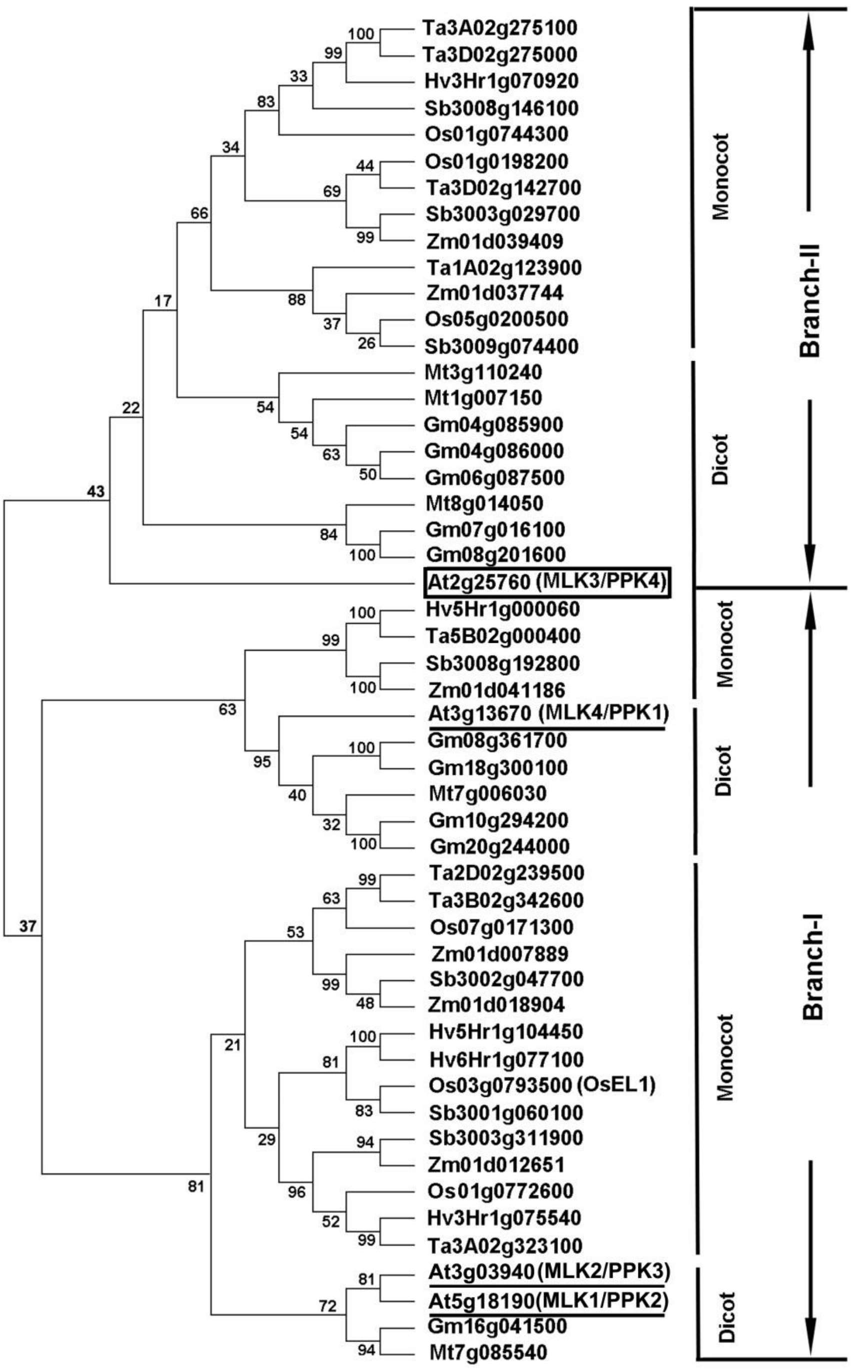

Figure S1. Homology analysis of the plant-specific CK1s in the indicated higher plants. MLKs and the ortholog proteins from seven crops were clustered using DNAMAN (version 7.0) with the default parameters. The numbers at the nodes of the neighbor-joining tree indicate the bootstrapping values based on 1000 interactions. MLK3 was boxed, and other three MLKs were underlined. For plant species: *Arabidopsis thaliana* (At), *Glycine max* (Gm), *Hordeum vulgare* (Hv), *Medicago truncatula* (Mt), *Oryza sativa* (Os), *Sorghum bicolor* (Sb), *Triticum aestivum* (Ta), and *Zea mays* (Zm). The accession number is from EnsemblPlants (https://plants.ensembl.org/Sorghum_bicolor/Info/Index).

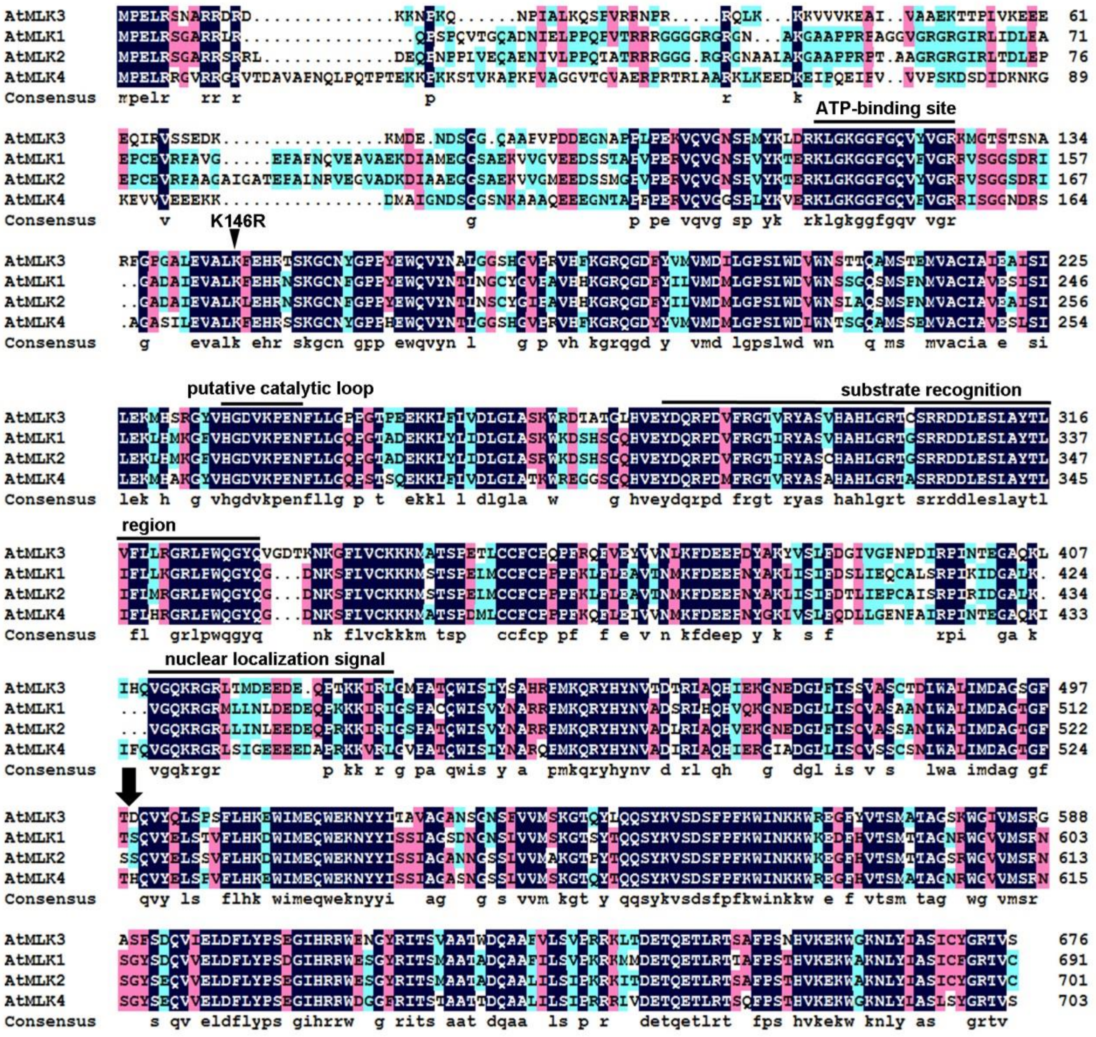

Figure S2. Sequence alignment of MLK1-4 in Arabidopsis. The point mutated lysine 146 was indicated by arrowhead and the interruption caused by T-DNA insertion in *mlk3* was indicated by arrow.

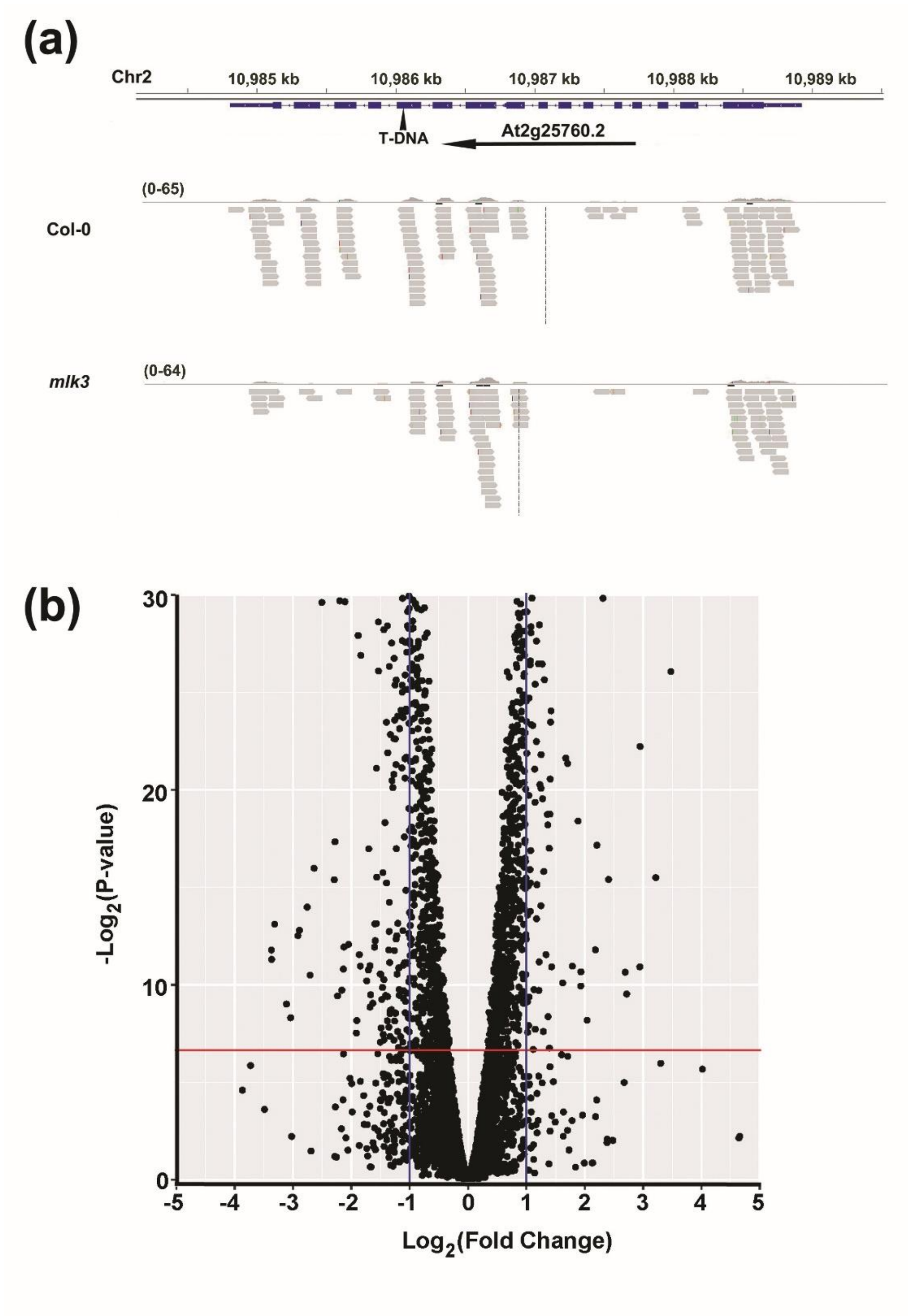

Figure S3. Transcriptome analysis of *mlk3* mutant relative to wild type. **(a)** The distribution of unique reads on *MLK3* (*At2g25760*) locus from RNA-sequencing. The upper and lower tracks represent wild type and *mlk3* mutant, respectively. The numbers indicate the coverage of reads range on the y-axis. Arrowhead indicates the T-DNA insertion site in *mlk3*, arrow for the forward strand of *MLK3* transcript. (**b**) Volcano plot of the differentially expressed genes. The horizontal line (in red) indicates the significance threshold (*P*=0.01) and the vertical lines (in blue) indicate the two fold change in transcript abundance threshold.

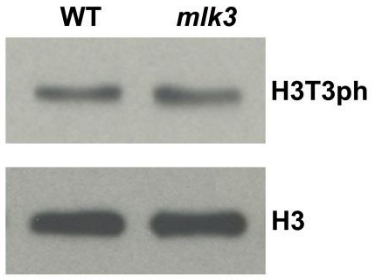

Figure S4. Analysis of H3T3ph level by western blot. Leaves from two-week-old plants were used for protein isolation. Immuno-blotting was conducted using antibody against H3T3ph with anti-H3 antibody (Abcam, ab1791) as an internal loading control.

Table S1. Sequences of the primers used in the study.

Usage	Primer sequence
Genotyping	LBa1: 5’- TGGTTCACGTAGTGGGCCATCG-3’
	P1: 5’-TGGATCAGCATTTACAGTGCTCACAGAC-3’
MLK3 RT-PCR	P2: 5’-TCCACTTGAAGGGAAAAGAATCACTGAC-3’
	P3: 5’- AGAACCCAATTGCTTTGAAACAATCA-3:
	P4: 5’- TCTGTGAGCACTGTAAATGCTG-3’
Actin2	F: 5’-CTAAGCTCTCAAGATCAAAGGC-3’
	R: 5’- AACATTGCAAAGAGTTTCAAGG-3’
35S::MLK3/	F: 5’-GGATCCATGCCTGAGCTGCGTAGCAACGCAC-3’
MLK3(K146R)	R: 5’-GAGCTCTCATGACACAGTTCGACCATAAC-3’
Point mutation	F: 5’-GGAGGTGGCTTTGAGGTTTGAGCATAGAACCAGC-3’
	R: 5’-GCTGGTTCTATGCTCAAACCTCAAAGCCACCTCC-3’
MLK3-GFP	F: 5’-CACCATGCCTGAGCTGCGTAGCAAC-3’
	R: 5’-TGACACA GTTCGACCATAACAA-3’
MLK3-MBP	F: 5'-GCGGCCGCATGCCTGAGCTGCGTAGCAACGCACGC-3’
	R: 5'-GGATCCTGACACAGTTCGACCATAACAAATCGATGC-3’
FLC	F: 5’-TTCTCCAAACGTCGCAACGGTCTC-3’
	R: 5’-GAGCTTTTGACTGATGA-3’
Ubiquitin 10	F: 5’- AGGATGGCAGAACTCTTGCT-3’
	R: 5’-TCCCAGTCAACGTCTTAACG-3’

Table S2. Basic information of the deduced MLK1-4 proteins in Arabidopsis.

	MLK1	MLK2	MLK3	MLK4
**No. a.a.**	691	701	676	703
**MW (kDa)**	76.98	77.99	76.49	78.64
**IP**	9.09	9.17	9.38	9.66
**CDS (bp)**	2076	2106	2031	2112
**Chromosome**	5	3	2	3

Table S3. Flowering time analysis of *mlk3* under short-day conditions.

Genotype	WT	*mlk3*
DAG	**58.2** ± 4.2	58.6 ± 4.1
No. Leaf	23.8 ± 2.9	24.1 ± 2.6

Note: data represent mean ± standard deviation (SD) of 10 plants under short-day conditions (8 h light/ 16 h dark). Days after germination (DAG) was measured as the number of days from germination to emergence of the floral bud, or the number of rosette leaves produced upon flowering was counted.
